# Periodontal Care as a Fundamental Step for an Active and Healthy Ageing

**DOI:** 10.1155/2013/127905

**Published:** 2013-12-17

**Authors:** Carlo Cafiero, Marco Matarasso, Gaetano Marenzi, Vincenzo Iorio Siciliano, Loredana Bellia, Gilberto Sammartino

**Affiliations:** University of Naples Federico II, Via Sergio Pansini 5, 80131 Napoli, Italy

## Abstract

In the industrialized part of the world, an increasing number of people live the old age without too many restrictions due to illness or physiological impairment. This group is known as *the young elderly*. On the contrary, a consistent part of seniors develops a greater number of medical conditions and become more and more dependent, these are *the old elderly*. The first cause of tooth lost in industrialized word is periodontitis that generally strikes people older than 40 years and determines serious detriment of the stomatognatic organ. Smoking and stress are risk factors for periodontitis that are common and shared between young, adult, and older age. Diabetes mellitus, obesity, and osteoporosis are very frequent pathological situations in older age. They have been identified as cofactors in the progression of periodontitis. Many dental associations recognize the importance of continued research on oral fluids diagnostics and welcome the development of rapid point-of-care tests providing accurate measurements of clinically validated biomarkers. At present, well-studied molecules associated with host response factors and with derived tissue destruction mediators have been proposed as diagnostic biomarkers for periodontitis detected in the oral fluids.

## 1. Introduction

In the word, the elderly are an assorted population group whose number is growing at a considerably faster rate (1.9%) than the total population (1.2%). In the European Union (EU), the number of people aged 65+ will almost double over the next 50 years, from 85 million in 2008 to 151 million in 2060 [1]. Being elderly has been held to be synonym to entering the third age: it lasts until the onset of the fourth age, that is characterized by deterioration, dependence, and ultimately death [2].

In the industrialized part of the world, an increasing number of people live this segment of life without too many restrictions due to illness or physiological impairment. This group is known as *the young elderly*. On the contrary, a consistent part of seniors develops a greater number of medical conditions and become more and more dependent. These are *the old elderly *[3].

Indeed, many conditions contribute to the frailty of the elderly, that is a condition making them more vulnerable to unfavourable health outcomes. Malnutrition, poor hydration, reduced physical activity, osteoporosis, and cognitive decline all contribute to poor health status. These conditions contribute to the occurrence, or to the evolution, of chronic diseases that are often present at the same time, and require complex poly-therapy regimens. In this context, poor prescription adherence is an additional component of frailty. The rising number of older people is linked to the growth of demands for health services and finally to unsustainable medical costs [4, 5].

While increased longevity is a great achievement, it is also a formidable challenge for both public and private budgets. For this reason, enlarging as much as possible the *young elderly group* appears fundamental, in order to have a healthy population and to lower nationals budgets covering the costs of Public Health. The European Commission has identified “active and healthy ageing” as a major social challenge, common to all the European countries. The pilot *European Innovation Partnership on Active and Healthy Ageing (EIP-AHA) *aims to enable EU citizens to lead healthy, active, and independent lives while ageing, by improving the sustainability and efficiency of social and health care systems. The all-encompassing target of this pilot partnership will be to increase the average healthy lifespan/lifetime by two years, by 2020 [6].

Prevention and early diagnosis of frailty and functional decline, both physical and cognitive, in older people is part of European Innovation Partnership on Active and Healthy Ageing 2020 initiative [7]. *The old elderly *group represents what we generally mean with the expression “to be old”, that is, to be *frail*. One of the key determinants of frailty is malnutrition, that is very common in older people. Malnourished older people are at risk of experiencing fallings, prolonged hospitalization, postoperative complications, pressure ulcers, and death. Many causes of malnutrition have been proposed: metabolism not working properly, poverty, loss of taste sensations, mental depression, and difficulties in mastication of aliments. Periodontitis determines teeth loss and consequently difficulties in mastication of food. Furthermore, periodontitis represents a persistent, systemic inflammatory condition that contributes to the occurrence of acute events in the subjects with concurrent cardiovascular diseases.

For these reasons, we speculate that periodontitis might be considered as an additional risk factor for frailty in the elderly.

## 2. Periodontitis. A Severe Infection of the Oral Cavity

The first cause of tooth lost in industrialized word is periodontitis that generally strikes people older than 40 years. It is a genetically determined gram-negative anaerobic infection characterized by complex immune reactions to bacterial burden that determines formation of periodontal pockets subsequent connective attachment loss and bone resorption around the roots and might lead to tooth loss. Periodontitis is characterized by a cyclic progression in which a recurrent active phase (periodontal breakdown) is followed by a quiescence phase; appropriate therapy generally stops its progression. Periodontitis is classified into aggressive and chronic forms. Aggressive periodontitis (AP) is less common than the chronic form. Generally it affects younger patients causing rapid loss of attachment and bone destruction. The severity of periodontal tissue destruction is conflicting with the scarce amounts of microbial deposits. The reason of this destruction is the presence of elevated proportions of aggressive gram-negative bacteria, the phagocyte abnormalities and the hyperresponsive macrophage phenotype. Chronic periodontitis (CP) affects up to 50% of the global population, especially older patients. In most cases, the rate of progression of chronic periodontitis is slow, and the amount of periodontal tissue destruction is generally commensurate with subgingival calculus and plaque amounts. Both forms, Aggressive and Chronic, might determine severe destruction of periodontal tissues: for this reason periodontitis has to be definitely considered as a social disease, since it might determine serious detriment of stomatognatic organ, till reach the condition of edentulism [8].

### 2.1. Environmental Risk Factors for Periodontitis

Smoking and stress are risk factors for periodontitis that are common and shared between young, adult, and older age. Diabetes mellitus, obesity, and osteoporosis are very frequent pathological situations in older age. They have been identified as cofactors in the progression of periodontitis.

### 2.2. Smoking

Cigarette smoking is associated with a relative risk of developing periodontitis, ranging from 2.05 (95% CI 1.47–2.87) for light smokers increasing to 4.75 (95% CI 3.28–6.91) for heavy smokers. The negative effect of smoking is dose-dependent and cumulative and is associated with the recurrence of periodontitis after healing but during periodontal maintenance programme care [9–11]. Most studies have shown that in smokers less gingival bleeding is recorded than nonsmokers [12, 13]. This situation could be subsequent to the vascular constriction determined by the nicotine effect. Moreover, the effect of smoking on periodontitis involve plaque microbiota composition, that determines higher prevalence of dangerous microbiota species (such as AA comitans, T. forsythia, and P. gingivalis), and scarce immunological reaction subsequently to impaired neutrophils and lymphocytes local reactions, as well as impaired fibroblast funcion.

### 2.3. Stress

Stress is currently considered as a risk indicator for periodontal disease. Stressful life events could affect periodontal disease progression through (1) unhealthy behaviours (poor oral hygiene, increased tobacco smoking) and (2) pathophysiological factors (higher glucocorticoid and catecholamine levels) which affect bacterial, immunological, inflammatory, and hormonal profiles, leading to an increased susceptibility to periodontal disease [14–16].

### 2.4. Diabetes Mellitus

Currently, the percentage of diabetics is very high worldwide, and these numbers are increasing dramatically. In the United States 8.3% of the population have diabetes. Between these, 10.9 million, or 26.9% of all people age 65 years or older have diabetes. These numbers mean that about one person upon four in elder group is affected by diabetes [17].

Patients with insulin-dependent and noninsulin-dependent diabetes mellitus have been found to be equally at risk of periodontitis [18]. It has been claimed that periodontitis is the sixth complication of diabetes, together with retinopathy, nephropathy, neuropathy, macrovascular diseases and altered wound healing [19]. Diabetes mellitus is the only systemic disease positively associated with attachment loss with an odds ratio of 2.32 (95% confidence interval (CI) 1.17–4.60) [20]. Attachment loss and increased alveolar bone loss were found to be common in patients affected by uncontrolled diabetes (glycosylated hemoglobin ≥ 8 mg/L).

The increased prevalence of periodontitis among diabetic patients has been attributed to the thickness augmentation of endothelial basement membrane, determining microangiopathy, as well as to neutrophils impaired function, hyper secretion of cytokines such as interleukin-1, interleukin-6, tumor necrosis factor-alpha, and prostaglandin E2. In particular, interleukin-6 appears to selectively suppress insulin action in hepatocytes. Some authors presumed a two-way relationship in which periodontal therapy can improve metabolic control in diabetic patients [21]. A recent Cochrane review on the treatment of periodontal disease for glycaemic control in people with diabetes declared that further controlled studies are necessary to clarify the topic [22].

### 2.5. Obesity

Obesity is defined as an unhealthy excess of body fat, which increases the risk of medical illness and premature mortality [23] The prevalence of obesity is increasing in all age groups, including older persons, defined as those ≥65 years old [24]. With aging there is a greater relative increase in intra-abdominal fat than in subcutaneous fat. In addition, increases in intrahepatic fat in older persons are associated with insulin resistance [25]. It has been suggested that obesity is a strong risk factor for periodontal tissue destruction [26], since adipose tissue represents more than simple fat accumulation, it should be considered as an endocrine organ. It produces cytokines and hormones, collectively called adipokines or adipocytokines, which may play a key role in modulating periodontitis [27–31]. In addition, it was reported that maintaining a normal weight was associated with a poorer frequency of periodontitis [32, 33].

### 2.6. Osteoporosis

Due to its prevalence worldwide, osteoporosis is considered as a serious public health concern [34]. Currently it is estimated that over 200 million people worldwide suffer from this disease [35]. Approximately 30% of all postmenopausal women have osteoporosis in the United States and in Europe. At least 40% of these women and 15–30% of men will experiment fragility fractures in lifetime [36]. Ageing of populations worldwide will be responsible for a major increase of the incidence of osteoporosis in postmenopausal women [37]. Osteoporosis is a metabolic bone disorder characterized by the loss of bone mineral density, principally recorded in postmenopausal women. It has been proposed that osteoporosis could affect the alveolar bone leading to rapid resorption in periodontal women, determining a positive relationship between osteoporosis and clinical attachment loss [38]. Other studies showed negative or equivocal results [39]. Thus, the association between osteoporosis and periodontitis in humans remains weak and still debatable [40].

### 2.7. Active and Healthy Aging. Early Diagnosis of Periodontitis as a Pathway to Get Young Elderly

Periodontitis has to be considered as a social disease since it affects millions of people in Europe as well as in the USA in which 31% of the population exhibited mild forms of periodontitis, 13% displayed periodontitis of moderate severity, and 4% suffered from advanced periodontitis [41]. Currently, accurate clinical periodontal diagnosis can be performed by periodontists, since they have sufficient diagnostic tools to get a clear picture of the actual single patient's periodontal conditions. Diagnostic imaging and periodontal charting provide a complete description of the patient's periodontal condition. By the use of a high-resolution professional digital camera, the operator takes a series of five pictures (frontal, right lateral, left lateral, palatal and lingual sides) that provides a clear picture of the patient's mouth. A full-mouth X-ray series is an important diagnostic support in periodontal patients (14/16 periapical X-rays); it provides information on the height and configuration of the interproximal alveolar bone and the state of the patient's individual tooth from the crown to the tip of its root. Periodontal charting (full-mouth plaque score, full-mouth bleeding score, probing depth clinical attachment level, bleeding on probing, recessions, mobility, migration, halitosis) provides a complete picture of periodontal conditions of a single patient [42]. Probing pocket depth (PPD) allows an immediate evaluation of diseased sites (six sites for each tooth). It represents the distance from the gingival margin to the bottom of the gingival sulcus/pocket, at the mesiobuccal line angle, the midbuccal, the distobuccal line angle, the distolingual line angle, the midlingual, and the mesiolingual line.

The above described diagnosis is generally performed by periodontists, that are specialists. Unfortunately, most of the patients are visited by general dentists which do not provide such high level performances. Since early diagnosis of periodontitis is fundamental in order to stop at initial stage the progression of the disease, it appears necessary to organize a diagnostic platform allowing periodontal screening by general dentists. With the intention to detect periodontitis at an earlier stage, when it is easier to be treated successfully, several easy-to-use chair side tests have been proposed. The following points represent pioneeristic attempt to organize early diagnosis of periodontitis through the detection of bacterial species and biomarkers.

- Some microbiota are more important than others as etiological agents of periodontitis. Following the above criteria, the consensus report of the world workshop on periodontitis [43] identified three bacterial species for which sufficient data have accumulated as causative factors for periodontitis: Aggregatibacter actinomycetemcomitans, P. gingivalis, and Tannerella forsythia [44]. The consensus report stated that A. actinomycetemcomitans is most often found in aggressive (“early onset”) periodontitis, whereas P. gingivalis and T. forsythia are found more frequently in chronic (“adult-onset”) periodontitis. Moderate evidence to support an aetiological P. nigrescens, Parvimonas micra (formerly Micromonas micros and Peptostreptococcus micros), the Streptococcus intermedius complex and T. denticola. Finally, an initial evidence included on the list of probable periodontal pathogens E. corrodens, enteric rods, Pseudomonas species, Selenomonas species, and Staphylococcus species. For these reasons, a DNA-based chair side test (semiquantitative polymerase chain reaction, PCR) of subgingival plaque could be a valid support for early diagnosis of periodontitis. Sampling of subgingival plaque by the insertion of sterile paper points into the deepest pockets in each quadrant are collected and send to a specialised laboratory that will perform the DNA examination and identification of bacterial species.
- In 1997, Kornman et al. described a composite genotype formed by two polymorphic loci—interleukin-1A(−889) and interleukin-1B (+3954)—which are single-nucleotide polymorphisms that carry a C-T transition. Interleukin-1 is a proinflammatory agent that is released by macrophages, lymphocytes, platelets, and endothelial cells. A sample of cells desquamating from the mucosa of the mouth allows examination to detect IL-1 polymorphism. A weak association between the single nucleotide Polymorphism in interleukin-1 genes and chronic periodontitis was found in a recent meta-analysis [45].
- Many dental associations, such as the American Dental Association (ADA), recognise the importance of continued research on oral fluids diagnostics and welcome the development of rapid point-of-care tests providing accurate measurements of clinically validated biomarkers. At present, well-studied molecules associated with host response factors and with derived tissue destruction mediators have been proposed as diagnostic biomarkers for periodontitis detected in the oral fluids. These components fall into three general categories: (1) host derived enzymes and their inhibitors, (2) inflammatory mediators, and host response modifiers and (3) tissue breakdown products.

We have searched the literature for more promising biomarker in regard to potential diagnostic value for periodontitis [46]. Consider the following.

- *Alkaline phosphatase.* Elevated alkaline phosphatase levels preceded periodontal destruction.
- *Beta-glucuronidase.* It could be thought as an indicator of periodontal disease activity. Nakashima [47] reported that beta-glucuronidase was significantly higher in active versus inactive sites.
- *Cathepsin B.* It is an enzyme active in proteolysis. Macrophages are the cellular source of cathepsin B in gingival crevicular fluid [48]. Cathepsin B levels (1) have been found to be increased in periodontitis but not in gingivitis [49–51].
- *Metalloproteinases-8.* It appears 18-fold higher in progressing periodontitis versus stable periodontitis [52].
- *Metalloprotheinases-9.* It appears elevated in subjects affected by advanced periodontitis associated with red complex anaerobic periodontal pathogens (e.g., P. gingivalis and T. denticola) [53].
- *Dipeptidyl peptidases II and IV.* Higher levels of both enzymes in sites with rapid and gradual attachment loss were reported with respect to sites without attachment loss [54].
- *Elastase.* It has been recorded in oral fluid from periodontal patients at elevated levels and reduced after periodontal treatment [55–57].
- *RANKL/OPG/RANK system*. In the course of periodontitis, RANKL is secreted by osteoblasts, fibroblasts, bone marrow stromal cells, and activated T and B cells.
- *Pyridinoline cross-linked carboxyterminal telopeptide of type I collagen.* High levels of 1-CTP were strongly correlated with clinical parameters and putative periodontal pathogens. Results showed that 1-CTP appeared as a good predictor of future alveolar bone and attachment loss and demonstrated significant reductions after periodontal therapy [58].
- *Chondroitin-4-sulphate (C-4-S).* It is the most common glycosaminoglycan in untreated chronic periodontitis [58]. A statistically significant correlation between the GCF content of C-4-S, a bone-specific glycosaminoglycan, and periodontal tissues destruction has been reported [59].

Even as gingival crevicular fluid (GCF), an exudate flushing from the gingival sulcus (0.5 to 2.5 mL/24 h) appears as the most appropriate diagnostic medium to use in analyses, it appears clear that the use of whole saliva is more practical even if reactants need to be highly sensitive since biomarkers are more diluted [60, 61].

## 3. Conclusions 

More Europeans are surviving into old age. It appears inevitable that the longer people live, the more their capacity for self-care become reduced by physical or mental chronic diseases.

In order to gain *active and healthy aging*, it appears essential to create favorable condition allowing better access to health care in the younger years and consequently postponing the period of dependence to advanced age, so that the period with disability will be compressed [62].

It is not easy to reach this objective. As described above, smoking, stress, diabetes, obesity and osteoporosis represent risk factors for both general and periodontal health.

Oral fluid is the mirror of periodontal health. It is a medium for clinically relevant information since it contains biomarkers specific for periodontal diseases. Progresses in microfluidics technology are revolutionizing molecular biology procedures for enzymatic analysis, DNA analysis and proteomics. The evolution of microfluidics, digital microfluidics, appears promising for future application to diagnose periodontal diseases and to prognosticate periodontal treatment. Lab-on-a-chip (LOC) technology for periodontal inspection will involve less education than current diagnostic procedures and allow patients to be screened for periodontal disease in settings other than the periodontist practice, such as at general practitioners, general dentists or dental hygienists. All these benefits make the lab-on-a-chip technology ideal for predictive, preventive, personalized, and participatory periodontology recently defined by a paper as “the 5Ps age” [8]. On these basis, we speculate that periodontists, dentists and medical general practitioners should all together make an effort in order to remove risk factors for periodontal and general health. For a very long time dental operators have considered their patients as “big mouths moving on tiny legs”. This wrong belief has led dentistry to abandon “mother house” general medicine and neglect fundamental aspects in diagnosis and therapy of several systemic disorders. Oral health has to be definitely considered as an important part of the human organism and not as an isolated segment. This appears like a “returning home” for dentistry. Finally, dental care is an important topic for healthy aging since a health stomatognatic apparatus is fundamental to get a good quality of life; first of all a good mastication means a good digestion and secondary food is an unequivocal mean helping in socialization of people, and socialization is a very important topic in old age for many reasons. Dental target of the immediate future is to allow elder people to arrive to advanced age with as much as possible functioning teeth in their mouth: this is the simple meaning of *active and healthy aging* in dentistry [63–65].
